# Arsenic in Subnitrate of Bismuth

**Published:** 1878-06

**Authors:** J. H. Salisbury

**Affiliations:** Chicago


### ARSENIC IN SUBNITRATE OF BISMUTH.

By J. H. Salisbury, M. D., Chicago.

It is well known that the subnitrate of bismuth is apt to contain arsenic as an impurity, but the frequency with which it occurs is probably not fully appreciated.

With a view to determining the frequency of this contamination of the preparations of bismuth, and the relative quantity which the specimens ordinarily sold contain, I undertook some investigations at the laboratory of Rush Medical College, under the direction of Prof. W. S. Haines.

Eighteen specimens of the subnitrate of bismuth, bought at different drug stores were examined. All the specimens but two were from reputable manufacturers—the names of the manufacturers of these two preparations were not known. Arsenic was found in thirteen of these. The quantity was determined in three and found to be 1-14 per cent. in the first; 1-10 per cent. in the second and 1-5 per cent. in the third.

Five specimens of subcarbonate of bismuth were examined and arsenic was found in all.

This result was quite unexpected as the subcarbonate is preferred by some to the subnitrate, as being less likely to contain arsenic on account of the mode of its preparation. My analyses would seem to show that this opinion is unfounded.

The arsenic in subnitrate of bismuth exists in the form of arseniate of bismuth—the percentages given above are calculated as arsenic acid.

The method given in the U. S. Dispensatory, p. 1065, for freeing the subnitrate from arsenic by boiling twice successively with potassic hydrate, dissolving the resulting oxide of bismuth in nitric acid, and reprecipitating with water, is effectual if carefully performed, as one of my experiments showed, in which I examined a specimen and found arsenic and examined the same specimen after purification by this process and found none. In another case however the process was less successful, and the subnitrate showed the presence of arsenic after attempted purification.

Arsenic in the preparations of bismuth is liable to be a dangerous impurity. Two cases are given by Taylor on Poisons, p. 470.

In one, puffiness of the eyes and gastro-intestinal irritation followed the taking of subnitrate of bismuth which was shown by analysis to contain a formidable proportion of arsenic. In another, a child showed symptoms of poisoning from subnitrate of bismuth given to control diarrhœa. The bismuth was shown to contain arsenic.

The largest quantity found in my analyses would amount to nearly $\frac{1}{8}$ of a grain in a drachm. This might produce very serious results if the subnitrate were given to a child or even to an adult, in the large doses often recommended to contol vomiting or diarrhœa.

The impurity is more dangerous from the fact that subnitrate of bismuth is usually regarded as a harmless drug, and hence is likely to be prescribed in large doses.

It seems very likely that this impurity when existing in quantity insufficient to produce poisonous effects, may counteract the sedative effects of the bismuth and thus render the medicine comparatively worthless.

It is an interesting question whether the good effects of subnitrate of bismuth in chronic gastric catarrh and in vomiting of various kinds, may not be due, to a certain extent, to the arsenic which it contains. Drop doses of Fowler's solution are recommended by Bartholow for chronic gastric catarrh and for some kinds of vomiting.

If subnitrate of bismuth containing 1-10 per cent. of arsenic were given, ten grains would contain an amount rather more than equivalent to a drop of Fowler's solution.

If the arsenic has in this way any beneficial effect it would be much better to have the preparations of bismuth pure, and to use in conjunction small doses of some preparation of arsenic so that definite doses could be given. Perhaps the arseniate of bismuth would be found to be an efficient therapeutic agent for this purpose.

In conclusion it is evident that sufficient care is not taken to free the preparations of bismuth from this dangerous impurity, so that the physician may know precisely what he is giving.

---

COLORLESS TINCTURE OF IODINE.—A mixture of tincture of iodine and carbolic acid will gradually produce tri-iodophenol, which is soluble in the alcohol. Hence the disappearance of color. The ingredients generally used are : ℞. tinct. iodinii comp. *m* xlv. ; acid. carbolic, *m* vj. ; glycerini fl. ℨ i ; aquæ fl. ℨ v. ; M. This is sometimes called carbolate of iodine. The color disappears in from eight hours to ten days.—*Can. Med. Record.*

